# Supplementary material for: Excised DNA circles from V(D)J recombination promote relapsed leukaemia
Source: Nature. 2025 Aug 6;645(8081):774–83. doi: 10.1038/s41586-025-09372-6 (PMC12443594; doi:10.1038/s41586-025-09372-6)
Supplement: Supplementary file 1 — This file contains Supplementary Figs. 1–4 [file 41586_2025_9372_MOESM1_ESM.pdf]

nature portfolio

https://doi.org/10.1038/s41586-025-09372-6

Supplementary information

Excised DNA circles from V(D)J recombination promote relapsed leukaemia

In the format provided by the authors and unedited

Supplementary Information

Excised DNA Circles from V(D)J Recombination Promote Relapsed Leukaemia

Zeqian Gao, James N.F. Scott, Matthew P. Edwards, Dylan Casey, Xiaoling Wang, Andrew D. Gillen, Sarra Ryan, Lisa J. Russell, Anthony V. Moorman, Ruth de Tute, Catherine Cargo, Anthony M. Ford, David R. Westhead, Joan Boyes

Legends to Supplementary Tables

Supplementary Table 1
SJs detected in WGS of *ETV6::RUNX1+* patients in the EGA database (EGA: EGAD00001000116).

Supplementary Table 2
The number of sequencing reads across each coding junction, as determined by LAM-recombination is shown. The patient numbers are given in each tab. R and NR denote patient samples taken at diagnosis for patients who did, and who did not, later relapse, respectively. Rel denotes patient samples taken at relapse. The final tab compares normalised LAM-recombination reads between diagnosis and relapse.

Supplementary Table 3
The number of sequencing reads across each signal joint, as determined by LAM-ESC is shown. SJs resulting from inversional recombination events were removed from all plots and analyses. LAM-ESC amplifies SJs by priming from J regions and therefore SJs resulting from intra-KV recombination[34] will not be detected. The patient numbers are given in each tab. R and NR denote patient samples taken at diagnosis for patients who did, and who did not, later relapse, respectively. Rel denotes patient samples taken at relapse.

Supplementary Table 4
The BCP-ALL subtype is shown for each patient used in the analyses. Relapse and No Relapse denote patient samples taken at diagnosis for patients who did, and who did not, later relapse, respectively. BM blasts shows the percent of leukaemic cells at diagnosis.

Supplementary Table 5
List of oligos used by type of experiment.

Supplementary Table 6
Summary of cohorts and source data used in each Figure.

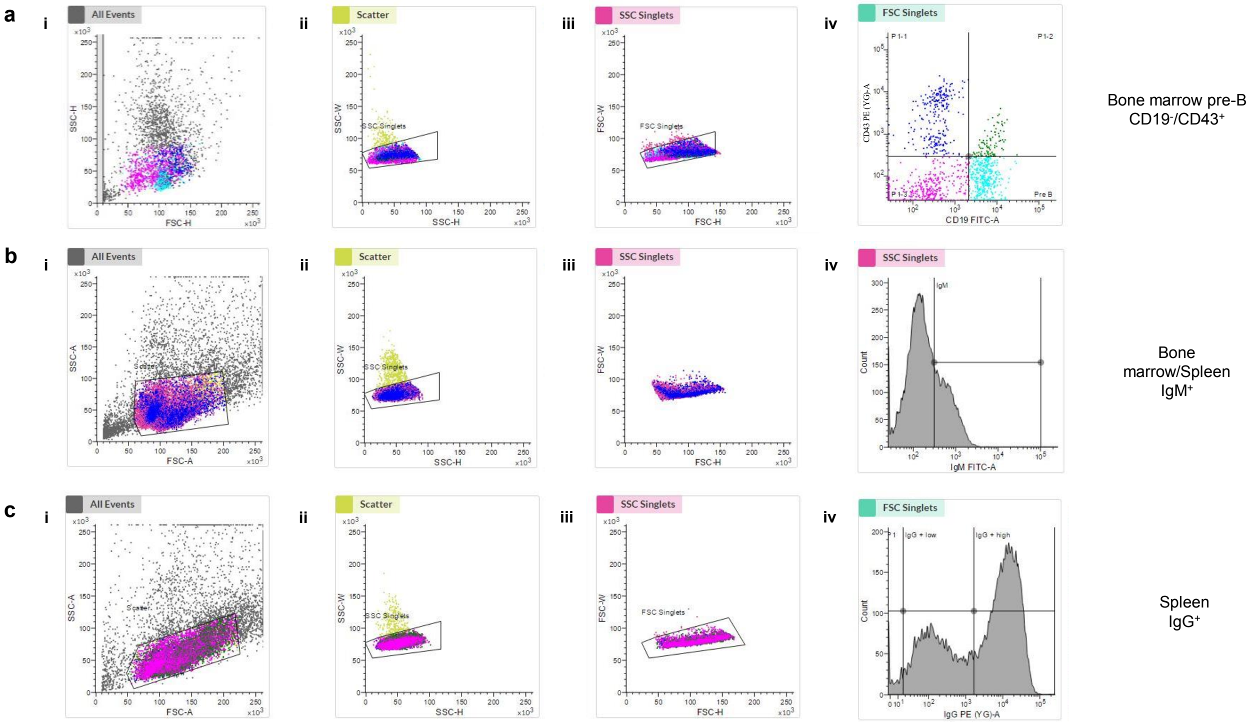

Supplementary Figure 1

Supplementary Fig. 1

Gating strategy to purify primary mouse B-cells. **a,** Bone marrow pre-B cells **(i)** Lymphocytes are gated on forward scatter (FSC-A) and side scatter (SSC-A). **(ii)** Single lymphocytes are gated on side scatter (SSC-H and SSC-W). **(iii)** Single lymphocytes are gated on forward scatter (FSC-H and FSC-W). **(iv)** Pre-B cells ($CD19^+/CD43^-$) are gated based on staining with FITC anti-CD19 and anti-CD43. **b,** Bone marrow / Spleen IgM^+. **(i-iii)** as for **(a)**. **(iv)** IgM^+ cells are gated based on staining with FITC anti-IgM. **c,** Spleen IgG^+. **(i-iii)** as for **(a)**. **(iv)** IgG^+ cells are gated based on staining with PE anti-IgG.

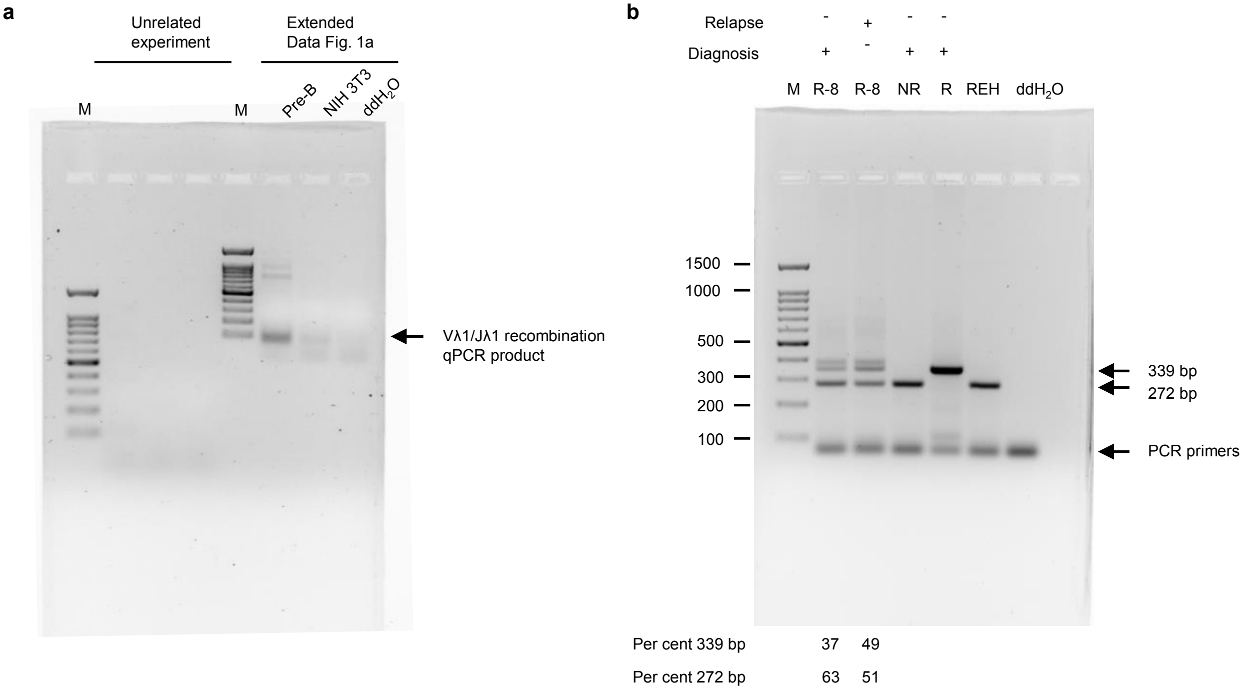

Supplementary Figure 2

Supplementary Fig. 2

Source images for the gels. Left: Gel for Extended Data Fig. 1a. The marker (PCRBio ladder IV) is shown in lane 5. Samples were taken from the qPCR experiment and run directly on the gel (lanes 6-8). Lanes 1-4 show an unrelated experiment. **b,** Right: Gel for Extended Data Fig. 9e. The marker (PCRBio ladder IV) is shown in lane 1. R and NR indicate samples taken at diagnosis from patients who did, and who did not, later relapse. ddH_2O indicates no template control. Quantification of the bands performed using AIDA image analysis software, is shown.

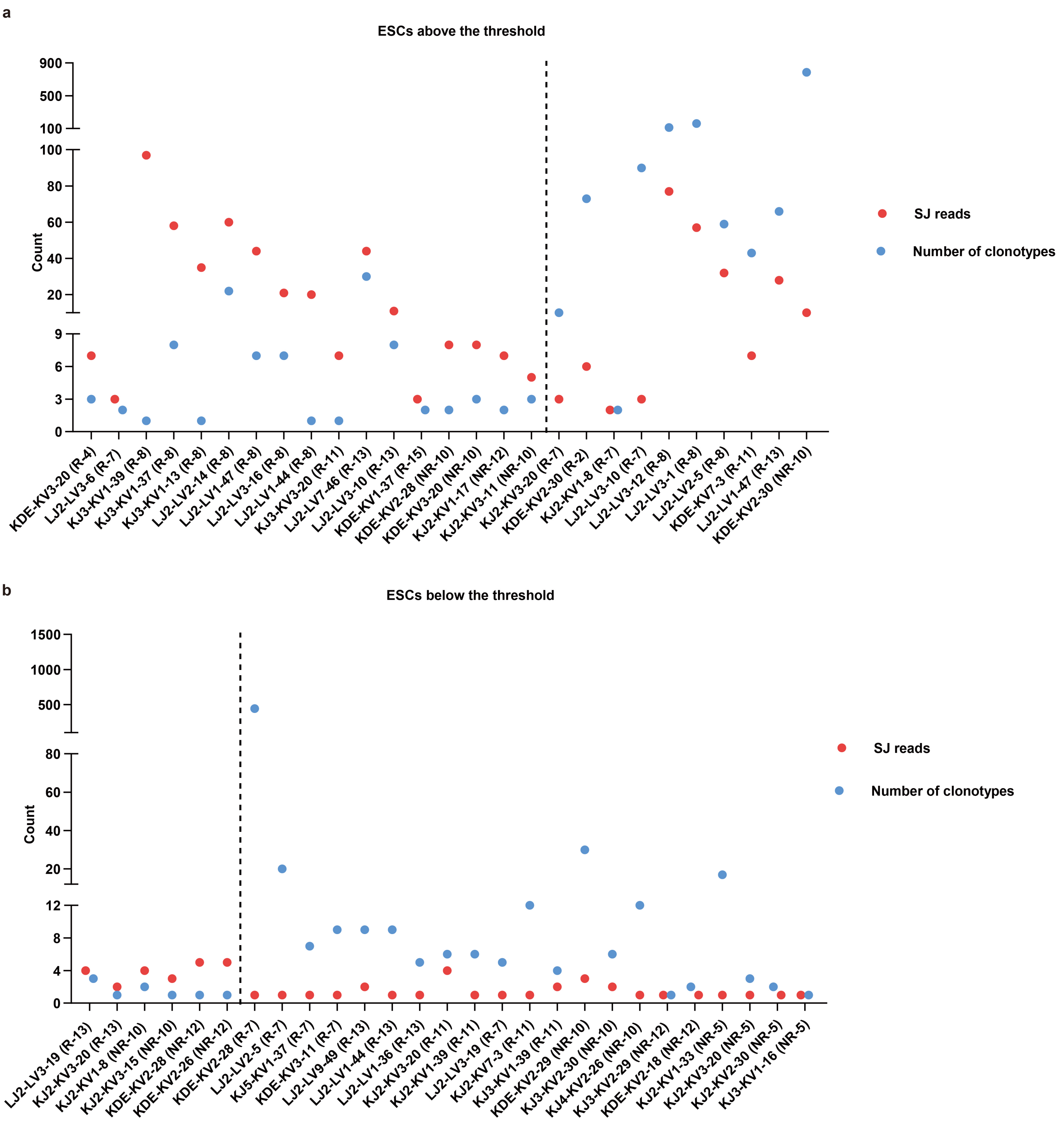

Supplementary Figure 3

Supplementary Fig. 3

Clonotype analysis of SJs. Comparison of individual clonotypes (from LAM-recombination reads) versus SJ reads (from LAM-ESC) for SJs above the threshold (upper; N=27) or below the threshold (lower; N=27), in LAM-ESC plots in Fig. 2a. SJ reads are given in red and the total number of clonotypes for the corresponding recombination in blue. The dotted line separates SJs where the reads exceed the clonotypes from those that do not. Sequence reads for which a clonotype could not be determined *via* IgBLAST (e.g. non-identifiable CDR3 sequences or recombination to KDE that is not detected by IgBLAST) were counted as unique clonotypes.

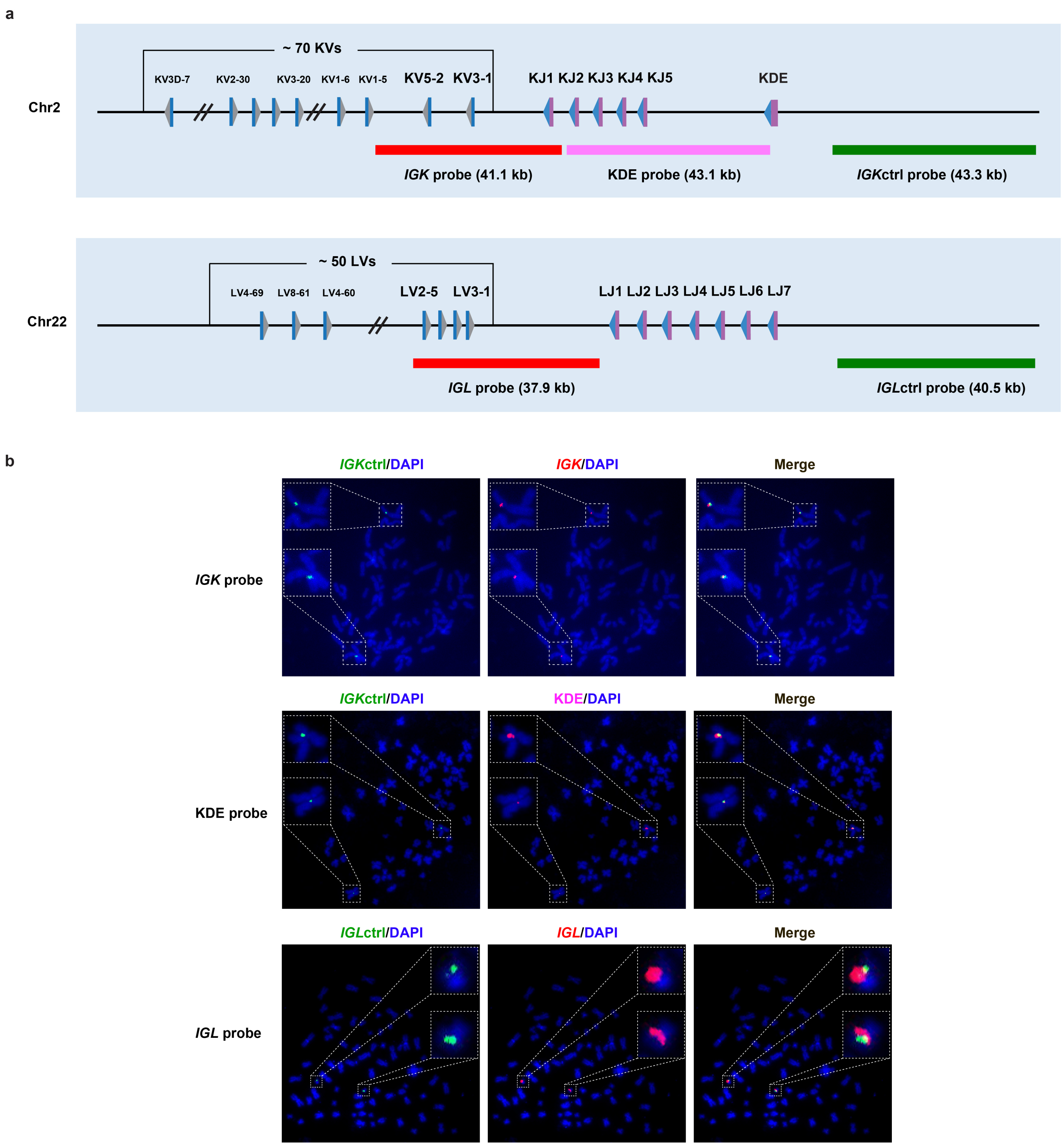

Supplementary Figure 4

Supplementary Fig. 4

Verification of FISH probes (a) Schematic of *IGK* and *IGL* loci indicating FISH probe locations. Probes are labelled as follows: Non-excised control regions (green): Alexa Fluor 488; regions excised to generate *IGK* and *IGL* ESCs (red): Alexa Fluor 555 and *IGK* KDE ESCs (pink): Alexa Fluor 647. (b) Hybridisation to metaphase spreads from hTERT-RPE-1 cells, with a normal diploid (female) karyotype. Chromosomes are stained with DAPI (blue). Upper: Hybridisation to *IGK* control (green) and ESC probe (red). Middle: Hybridisation to *IGK* control (green) and KDE ESC probe (pink). Lower: Hybridisation to *IGL* control (green) and ESC probe (red). Magnification: 100X.
