# Supplementary material for: Excised DNA circles from V(D)J recombination promote relapsed leukaemia
Source: Nature. 2025 Aug 6;645(8081):774–83. doi: 10.1038/s41586-025-09372-6 (PMC12443594; doi:10.1038/s41586-025-09372-6)
Supplement: Supplementary file 2 — Reporting Summary [file 41586_2025_9372_MOESM2_ESM.pdf]

nature portfolio

Corresponding author(s): Joan Boyes

Last updated by author(s): Jun 23, 2025

Reporting Summary

Nature Portfolio wishes to improve the reproducibility of the work that we publish. This form provides structure for consistency and transparency in reporting. For further information on Nature Portfolio policies, see our Editorial Policies and the Editorial Policy Checklist.

Statistics

For all statistical analyses, confirm that the following items are present in the figure legend, table legend, main text, or Methods section.

n/a	Confirmed	
☐	☒	The exact sample size (*n*) for each experimental group/condition, given as a discrete number and unit of measurement
☐	☒	A statement on whether measurements were taken from distinct samples or whether the same sample was measured repeatedly
☐	☒	The statistical test(s) used AND whether they are one- or two-sided<br>*Only common tests should be described solely by name; describe more complex techniques in the Methods section.*
☒	☐	A description of all covariates tested
☐	☒	A description of any assumptions or corrections, such as tests of normality and adjustment for multiple comparisons
☐	☒	A full description of the statistical parameters including central tendency (e.g. means) or other basic estimates (e.g. regression coefficient) AND variation (e.g. standard deviation) or associated estimates of uncertainty (e.g. confidence intervals)
☐	☒	For null hypothesis testing, the test statistic (e.g. *F*, *t*, *r*) with confidence intervals, effect sizes, degrees of freedom and *P* value noted<br>*Give P values as exact values whenever suitable.*
☒	☐	For Bayesian analysis, information on the choice of priors and Markov chain Monte Carlo settings
☒	☐	For hierarchical and complex designs, identification of the appropriate level for tests and full reporting of outcomes
☐	☒	Estimates of effect sizes (e.g. Cohen's *d*, Pearson's *r*), indicating how they were calculated

Our web collection on statistics for biologists contains articles on many of the points above.

Software and code

Policy information about availability of computer code

Data collection

FISH images were acquired using Olympus CellSens Dimension 3.2 (Build 23706) software
Flow cytometry data were collected using BDFACSChorus 3.0, version 1.4.3.0
ddPCR data was acquired and analysed using the Bio-Rad QuantaSoft v1.7.4 software
qPCR data was acquired and analysed using Corbett Rotor-Gene 6000 Series Software (v.1.7, build 87).

Data analysis

A custom Python script to analyse SJs in WGS datasets (Extended Data Fig. 2e) is available at (https://github.com/Boyes-Lab/NGS-Analysis; DOI: 10.5281/zenodo.15412502).
A custom Python script to analyse LAM-ESC and LAM-recombination data (Fig. 2 and others), together with a custom database is available at https://github.com/Boyes-Lab/LAM-ESC-Recombination; DOI: 10.5281/zenodo.15412315. The script automates BLAST searches against a custom BLAST database, consisting of all V-J recombination events or all head-to-head RSS combinations from the immunoglobulin kappa and lambda loci, for recombination and SJ libraries, respectively.
Differentially expressed genes were identified using DESeq2 with |logFC| > 0.585 and FDR < 0.05. Gene set enrichment analysis (GSEA) was carried out according to the user guide provided by the BROAD Institute (https://docs.gsea-msigdb.org/#GSEA/GSEA_User_Guide/).
In silico experiments to detect ecDNAs were carried out using AmpliconArchitect according to the AmpliconSuite pipeline (https://github.com/AmpliconSuite/AmpliconSuite-pipeline/blob/master/documentation/GUIDE.md).
A custom Python script was used to determine the clonotypes present in sequencing reads compared to the reference motifs near the breakpoint junction of interest (Extended Data Fig. 7b). Clonotype_analysis.py, is available at: (https://github.com/Boyes-Lab/NGS-Analysis; DOI: 10.5281/zenodo.15412502).
A bespoke Python programme to analyse breakpoints of SVs, SVs_near_RSSs.py, (Fig. 5a, b) is available at (https://github.com/Boyes-Lab/Structural-Variants; DOI: 10.5281/zenodo.15412565). This creates an analysis window spanning 50 bp either side of each breakpoint; the

presence of an RSS within the window is then analysed using the DNAGrep algorithm, via RSSite.
A custom Python script to analyse potential cut-and-run events compared to reintegration events is provided at https://github.com/Boyes-Lab/Structural-Variants; DOI: 10.5281/zenodo.15412565.
Python version 3.10 was used for all Python scripts.
FISH images were analysed using FIJI 2.16.0 software.
Agarose gels were quantified using FIJI Image J 2, version 2.14.0/1.5f

For manuscripts utilizing custom algorithms or software that are central to the research but not yet described in published literature, software must be made available to editors and reviewers. We strongly encourage code deposition in a community repository (e.g. GitHub). See the Nature Portfolio guidelines for submitting code & software for further information.

Data

Policy information about availability of data

All manuscripts must include a data availability statement. This statement should provide the following information, where applicable:

- Accession codes, unique identifiers, or web links for publicly available datasets
- A description of any restrictions on data availability
- For clinical datasets or third party data, please ensure that the statement adheres to our policy

WGS datasets from 61 ETV6::RUNX1+ BCP-ALL patients were downloaded from the European Genome-phenome Archive (EGA), dataset ID: EGAD00001000116). The human genome sequence hg19, (GCA_000001405.14) release GRCh37.p13, was downloaded from https://hgdownload.soe.ucsc.edu/goldenPath/hg19/bigZips/. Structural variant data of BCP-ALL patients at diagnosis and relapse were downloaded from the Complete Genomics (CGI, from within the TARGET database: dbGaP Sub-study ID: phs000464). RNA-seq data of BCP-ALL patients at diagnosis were downloaded from the TARGET database (dbGaP Sub-study ID: phs000464). RNA-seq and WGS datasets from BCP-ALL patients in the VIVO Biobank cohort were downloaded from EGA under the Accession Code EGAS00001006863. Raw LAM-ESC and LAM-recombination sequences are available from the European Genome-phenome Archive (EGA), under the dataset ID: EGAD50000000597. The extracted recombination junctions and ESCs are given in Supplementary Tables 2 and 3. Whole exome sequencing data from BCP-ALL patients in the VIVO Biobank cohort are available from EGA via the dataset ID: EGAD50000001519. Amplicon sequencing data of the recombination junctions used for clonotype analysis are available from EGA under the dataset ID: EGAD50000001518. Source data for all graphs is available as three Excel spreadsheets for main, Extended Data and Supplementary Figures, respectively. FISH data are available via Research Data Leeds63: https://doi.org/10.5518/1693. The sample cohort used for each Figure is given in Supplementary Table 6.

Research involving human participants, their data, or biological material

Policy information about studies with human participants or human data. See also policy information about sex, gender (identity/presentation), and sexual orientation and race, ethnicity and racism.

Reporting on sex and gender	Sex- or gender-based analyses were not performed as the incidence of BCP-ALL is known to be similar between males and females (1:1.2). The study was initially performed blind. Information provided following data acquisition showed that very similar numbers of samples from males and females were used across both patient groups (i.e. patients known to subsequently relapse and those who remained in remission).
Reporting on race, ethnicity, or other socially relevant groupings	No socially constructed or socially relevant categorisation variables were used. Although there is a higher incidence of BCP-ALL in the upper hemisphere, the centres from which the samples were received were all from the upper hemisphere (UK and Czech Republic). Therefore, samples used in the study should not be subject to variables between higher and lower disease incidence areas.
Population characteristics	The human research participants are all under age 25 since the peak age group for BCP-ALL is children and young adults. WGS and RNA-seq data are also available for BCP-ALL patients in this age group, via EGA (dataset IDs: EGAD00001000116 and EGAS00001006863) and the TARGET dataset (NIH), allowing comparison of our data with big datasets. Relevant genotypic information regarding BCP-ALL subtype of the human participants is given in Supplementary Table 4. All human participants had been diagnosed with ALL, apart from normal blood samples from two control participants. Information on whether the participants with BCP-ALL suffered relapse disease is given in Supplementary Figure 4.
Recruitment	Patients were recruited to donate samples via leaflets/information provided to them by their clinicians. Donations to VIVO Biobank were from throughout the UK, typically from larger centres participating in ALL trials. Samples were donated to HMDS from throughout Northern England whereas those in the Czech Republic were from patients in one of the larger ALL treatment centres. Informed consent was obtained from the parents or legal guardians of children with ALL, eliminating self-selection bias.
Ethics oversight	Patient samples, taken as part of routine diagnostics, were supplied by VIVO Biobank, HMDS or a hospital in the Czech Republic. Collection and use of patient samples were approved by the appropriate institutional review board (IRB). Each organisation obtained informed patient consent for anonymised samples to be used by third parties for research. The use of surplus diagnostic material for research by HMDS and collaborators was approved by the Health Research Authority (HRA): 04_Q1205_125. Local ethics approval was obtained from the Biological Sciences Research Ethics Committee, University of Leeds: BIOSCI 18-031, 2308 and CCR 2285, Royal Marsden Hospital NHS Foundation Trust.

Note that full information on the approval of the study protocol must also be provided in the manuscript.

Field-specific reporting

Please select the one below that is the best fit for your research. If you are not sure, read the appropriate sections before making your selection.

☒ Life sciences ☐ Behavioural & social sciences ☐ Ecological, evolutionary & environmental sciences

For a reference copy of the document with all sections, see nature.com/documents/nr-reporting-summary-flat.pdf

Life sciences study design

All studies must disclose on these points even when the disclosure is negative.

Sample size	An initial power calculation was performed, based on preliminary data, to determine the number of samples that should be analysed to determine if the link between higher ESC levels and subsequent relapse, is statistically significant. Based on this, one hundred samples were requested from VIVO Biobank. Although sample quality precluded analysis of some samples, as many samples as feasible were analysed. Statistical tests were applied to the data to determine if sufficient samples had been tested.
Data exclusions	For LAM-ESC data, signal joints (SJs) involving KV gene segments that are known to undergo inversional recombination were excluded from further analyses. This is because inversional recombination results in SJs that are retained in the genome and therefore such SJs do not represent extra-chromosomal (ESC) DNA. This is stated in the legend to Supplementary Table 3 that shows the LAM-ESC data.
Replication	Reproducibility was confirmed by performing three technical repeats for each experiment. Three biological repeats were performed with mouse samples where this number of biological repeats is possible. For patient samples, reproducibility was confirmed using samples from at least three patients and often many more, depending on the number of samples that fitted the criteria of the experiment (such as ESCs at a given level or that appeared to persist from the primary recombination event). All attempts at replication were successful.
Randomization	Samples from BCP-ALL patients, taken at diagnosis, were provided with information about whether the patient subsequently relapsed. Otherwise, analyses were initially blinded. Experiments were performed in exactly the same way for all samples; data were analysed by comparing patients who later relapsed with those who did not. Further analyses of covariates once additional information was made available showed roughly similar numbers of males and females in each group and representatives of different BCP-ALL subtypes in each group. A large sample size was used to reduce the impact of other variables.
Blinding	Anonymised patient samples were blinded for all variables apart from whether or not the patient subsequently relapsed.

Reporting for specific materials, systems and methods

We require information from authors about some types of materials, experimental systems and methods used in many studies. Here, indicate whether each material, system or method listed is relevant to your study. If you are not sure if a list item applies to your research, read the appropriate section before selecting a response.

Materials & experimental systems

n/a	Involved in the study	
☐	☒	Antibodies
☐	☒	Eukaryotic cell lines
☒	☐	Palaeontology and archaeology
☐	☒	Animals and other organisms
☒	☐	Clinical data
☒	☐	Dual use research of concern
☒	☐	Plants

Methods

n/a	Involved in the study	
☒	☐	ChIP-seq
☐	☒	Flow cytometry
☒	☐	MRI-based neuroimaging

Antibodies

Antibodies used	FITC anti-CD19 (BD Pharmingen, #553785) Clone 1D3<br>PE anti-CD43 (BD Pharmingen, #553271). Clone S7. Lot 7172765<br>FITC anti-IgM (BD Pharmingen, #553408) Clone R6-60.2. Lot 31529<br>PE anti-IgG (eBioscience, #12-4010-82). Polyclonal. Lot 4315025<br>Anti-BrdU (BD Pharmingen #555627). Clone 3D4(RUO). Lot 4015735<br>Goat Anti-Mouse IgG (Jackson ImmunoResearch #115-001-003). Polyclonal. Lot 92114<br>Alexa Fluor 488 donkey anti-goat (ThermoFisher #A32814). Polyclonal. Lot VK308431
Validation	Validation and references are provided on the manufacturer's websites:<br><br>FITC anti-CD19 (BD Pharmingen, #553785) Clone 1D3<br>https://www.bdbiosciences.com/en-us/products/reagents/flow-cytometry-reagents/research-reagents/single-color-antibodies-ruo/fitc-rat-anti-mouse-cd19.553785

PE anti-CD43 (BD Pharmingen, #553271). Clone S7.
https://www.bdbiosciences.com/en-eu/products/reagents/flow-cytometry-reagents/research-reagents/single-color-antibodies-ruo/pe-rat-anti-mouse-cd43.553271

FITC anti-IgM (BD Pharmingen, #553408) Clone R6-60.2
https://www.bdbiosciences.com/content/dam/bdb/products/global/reagents/flow-cytometry-reagents/research-reagents/single-color-antibodies-ruo/553xxx/5534xx/553408_base/pdf/553408.pdf

PE anti-IgG (eBioscience, #12-4010-82). Polyclonal
https://www.thermofisher.com/antibody/product/Goat-anti-Mouse-IgG-H-L-Secondary-Antibody-Polyclonal/12-4010

Anti-BrdU (BD Pharmingen #555627). Clone 3D4(RUO).
https://www.bdbiosciences.com/en-gb/products/reagents/flow-cytometry-reagents/research-reagents/single-color-antibodies-ruo/purified-mouse-anti-brdu.555627?tab=product_details

Goat Anti-Mouse IgG (Jackson ImmunoResearch #115-001-003).
https://www.jacksonimmuno.com/catalog/products/115-001-003

Alexa Fluor 488 Donkey anti-goat (ThermoFisher #A32814). Polyclonal.
https://www.thermofisher.com/antibody/product/Donkey-anti-Goat-IgG-H-L-Highly-Cross-Adsorbed-Secondary-Antibody-Polyclonal/A32814

Eukaryotic cell lines

Policy information about cell lines and Sex and Gender in Research

Cell line source(s)	hTERT-RPE-1: ATCC. Derived from a diploid human female<br>NIH3T3: A gift from Professor Constanze Bonifer, University of Leeds. Fibroblast cell line derived from male mice.<br>HeLa: A gift from Professor Tariq Enver, ICR, London. Human cervical cancer cell line derived from a female.
Authentication	hTERT-RPE-1: Authenticated by ATCC by morphology, STR profiling and karyotyping; Lot number 70043063<br>NIH3T3: Authenticated by amplification with mouse-specific PCR primers<br>HeLa: Authenticated by amplification with human-specific PCR primers.
Mycoplasma contamination	hTERT-RPE-1: Tested by ATCC. Mycoplasma negative by Hoechst DNA staining, agar culture and a PCR-based assay. Not subsequently tested for mycoplasma<br>NIH3T3: Confirmed negative for mycoplasma using MycoAlert® Mycoplasma Detection Kit, Catalog No. LT07-318 in 2025<br>HeLa: Confirmed negative for mycoplasma using MycoAlert® Mycoplasma Detection Kit, Catalog No. LT07-318 in 2025
Commonly misidentified lines<br>(See ICLAC register)	HeLa cells have been misidentified as liver cells (Chang liver) where HeLa cells appear to have contaminated the liver cell line. Similarly, HeLa cell contamination has been reported for other cell lines, including HEp-2, KB and J111. In each case, HeLa cells contaminated the other cell lines. Given that we were only using DNA from HeLa cells as a source of non-B cell human DNA and the DNA hybridised as expected to human-specific PCR primers and the cells have the correct morphology, we did not test if another human cell line had been misidentified as HeLa cells.

Animals and other research organisms

Policy information about studies involving animals; ARRIVE guidelines recommended for reporting animal research, and Sex and Gender in Research

Laboratory animals	CBA/C57BL/6J mice between 5 and 7 weeks old were used
Wild animals	The study did not involve wild animals
Reporting on sex	Pre-B cells were isolated from the femurs of 12 CBA/C57BL/6J mice whereas spleens were isolated from three mice. All mice were 5-7 weeks old. The sex of the mice was not considered in the study design as this is unlikely to influence the B cells used. Instead, approximately equal numbers of male and female mice were used. These had been housed in a full barrier facility where animals are free from common pathogens.
Field-collected samples	The study did not involve samples collected from the field
Ethics oversight	Animal procedures were performed under Home Office licence P3ED6C7F8, following review by the University of Leeds ethics committee.

Note that full information on the approval of the study protocol must also be provided in the manuscript.

Plants

Seed stocks	No seed stocks were used
Novel plant genotypes	Plants were not used in this study
Authentication	Plants were not used in this study

Flow Cytometry

Plots

Confirm that:

☒ The axis labels state the marker and fluorochrome used (e.g. CD4-FITC).

☒ The axis scales are clearly visible. Include numbers along axes only for bottom left plot of group (a 'group' is an analysis of identical markers).

☐ All plots are contour plots with outliers or pseudocolor plots.

☐ A numerical value for number of cells or percentage (with statistics) is provided.

Methodology

Sample preparation	Mouse femurs and spleens were collected from 5 to 7-week-old mice. Bone marrow (BM) cells were flushed from femurs with PBS whereas splenocytes were prepared by flushing cells from finely diced pieces of spleen with PBS through a 50 µm cell strainer. Following preparation of single cell suspensions in PBS, BM cells and splenocytes were centrifuged at 600 x g for 3 minutes and resuspended in 10 ml of 0.168 M NH4Cl to lyse erythrocytes. After 10 minutes, cells were washed with 40 ml PBS and resuspended in 1 ml staining buffer (2% FCS, 1 mM EDTA, 25 mM HEPES-KOH pH 7.9 in PBS).<br>Cells were stained with the appropriate antibodies prior to purification by flow cytometry. For BM pre-B cells, 1 ml cell suspensions were stained with 6 µl each of FITC anti-CD19 (BD Pharmingen, #553785) and PE anti-CD43 (BD Pharmingen, #553271). BM or spleen IgM+ cells were stained with 10 µl FITC anti-IgM (BD Pharmingen, #553408 - 1 in 100 dilution) whereas spleen IgG+ cells were stained with 15 µl PE anti-IgG (eBioscience, #12-4010-82 - 3 in 200 dilution). Following incubation at room temperature for 10 minutes, cells were washed with PBS and resuspended in 0.5 ml staining buffer
Instrument	Purification was via a FACSMelody (BD) cell sorter
Software	BDFACSChorus software was used to run the flow cytometer and to collect the post-sort purity data
Cell population abundance	Post-sort sample purity was verified by running a sample of sorted cells through the FACSMelody. Only samples with >90% purity were used for further study.
Gating strategy	Gating strategies were as follows:<br>Bone marrow pre-B cells: (i) Lymphocytes were first gated on forward scatter (FSC-A) and side scatter (SSC-A). (ii) Single lymphocytes were then gated on side scatter (SSC-H and SSC-W). (iii) Single lymphocytes were further gated on forward scatter (FSC-H and FSC-W). (iv) Pre-B cells (CD19+/CD43-) were gated based on their staining with FITC anti-CD19 (CD19 FITC-A) and anti-CD43 (CD43 PE(YG)-A).<br>Bone marrow / Spleen IgM+ cells. Gating was as above for steps i-iii. (iv) IgM+ cells were gated based on their staining with FITC anti-IgM (IgM FITC-A).<br>Spleen IgG+ cells: Gating was as above for steps i-iii. (iv) IgG+ cells were gated (IgG + high) based on their staining with PE anti-IgG (IgG PE (YG)-A).

☒ Tick this box to confirm that a figure exemplifying the gating strategy is provided in the Supplementary Information.
