# Supplementary material for: Excised DNA circles from V(D)J recombination promote relapsed leukaemia
Source: Nature. 2025 Aug 6;645(8081):774–83. doi: 10.1038/s41586-025-09372-6 (PMC12443594; doi:10.1038/s41586-025-09372-6)
Supplement: Supplementary file 8 — Summary of cohorts and source data used in each figure. [file 41586_2025_9372_MOESM8_ESM.pdf]

Figure	Cohort/ Database used	Sample numbers	Data availability
2a and b	BCP-ALL patient samples from VIVO biobank (Relapse and Non-relapse)<br>Healthy blood (University of Leeds)<br>Remission sample (HMDS)	N = 34 (Relapse)<br>N = 37 (Non-relapse)<br>N = 2 (Healthy blood)<br>N = 1 (Remission)	LAM-recombination and LAM-ESC data available from European Genome-phenome Archive (EGA), under the dataset ID: EGAD50000000597.<br>Processed data available from Supplementary Tables 2 and 3.
2c	BCP-ALL patient samples from VIVO biobank	N = 5 (Relapse)<br>N = 6 (Non-relapse)	LAM-ESC data available from European Genome-phenome Archive (EGA), under the dataset ID: EGAD50000000597.<br>Processed data available from Supplementary Table 3.<br>Source qPCR data are available via the Source data Excel spreadsheet: Main figures
3b	BCP-ALL patient samples from VIVO biobank	N = 74 SJs (Relapse)<br>N = 51 SJs (Non-relapse)<br>N = 12 patients (Relapse)<br>N = 8 patients (Non-relapse)	LAM-recombination and LAM-ESC data, available from European Genome-phenome Archive (EGA), under the dataset ID: EGAD50000000597.<br>Processed data available from Supplementary Tables 2 and 3.
3c	BCP-ALL patient samples from VIVO biobank	N = 11 pairs of ESCs<br>N = 31 (Relapse)<br>N = 30 (Non-relapse)	Source ddPCR data are available via the Source data Excel spreadsheet: Main figures
3d	TARGET database; BCP-ALL patients	N = 74 (Relapse)<br>N = 49 (Non-relapse)	https://www.cancer.gov/ccg/research/genome-sequencing/target, dbGaP Sub-study ID: phs000464
3e	BCP-ALL patient samples from VIVO biobank	N = 11 in all cases except: *PCNA*, Relapse, low ESC: N = 8; *POLE3*, Relapse, low ESC: N = 10; *RBX1*, Non-relapse: N = 12.	Source qPCR data are available via the Source data Excel spreadsheet: Main figures
3f	BCP-ALL patient samples from VIVO biobank	N = 4 (high ESC levels)<br>N = 3 (low ESC levels)	RNA-seq data available from European Genome-phenome Archive (EGA) under the Accession Code EGAS00001006863.
4a	BCP-ALL patient samples from VIVO biobank	N = 40 (Relapse) from 23 patients<br>N = 25 (Non-relapse) from 20 patients	LAM-recombination and LAM-ESC data, available from European Genome-phenome Archive (EGA), under the dataset ID: EGAD50000000597.<br>Processed data available from Supplementary Tables 2 and 3.

4b	BCP-ALL patient samples from VIVO biobank	N = 23 high copy SJs from 7 patients<br>N = 124 low copy SJs from 12 patients	LAM-recombination and LAM-ESC data, available from European Genome-phenome Archive (EGA), under the dataset ID: EGAD50000000597.<br>Processed data available from Supplementary Tables 2 and 3.
4c	BCP-ALL patient samples from HMDS	N = 29, 24 and 26 images from 3 patients (M, N and P, respectively)	FISH data are available via Research Data Leeds: https://doi.org/10.5518/1693
4d	BCP-ALL patient samples from HMDS	N = 127, 101, and 108 images from 3 patients (M, N and P, respectively)	FISH data are available via Research Data Leeds: https://doi.org/10.5518/1693
4f	BCP-ALL patient samples from HMDS	Representative FISH image from N = 127, 101, and 108 images from 3 patients (M, N and P, respectively)	FISH data are available via Research Data Leeds: https://doi.org/10.5518/1693
4g	BCP-ALL patient samples from HMDS	Representative FISH image of cell with both *IGK* and *IGL* ESCs. N = 0 (Patient M); N = 3 (Patient N) and N = 7 (Patient P)	FISH data are available via Research Data Leeds: https://doi.org/10.5518/1693
4h	BCP-ALL patient sample from HMDS	Representative FISH image of cell with ≥ 3 ESCs. N = 8 from Patient P	FISH data are available via Research Data Leeds: https://doi.org/10.5518/1693
5a	TARGET database; BCP-ALL patients	N = 121 (Relapse)<br>N = 29 (Non-relapse)	https://www.cancer.gov/ccg/research/genome-sequencing/target, dbGaP Sub-study ID: phs000464
5b	TARGET database; BCP-ALL patients	N = 83 matched samples at diagnosis and relapse	https://www.cancer.gov/ccg/research/genome-sequencing/target, dbGaP Sub-study ID: phs000464
5c	LAM-HTGTS data	N = 4 independent LAM-HTGTS experiments	Data available as FASTQ files from NCBI SRA: PRJNA483469
5d and e	BCP-ALL patient samples from VIVO biobank	N = 10 recombination junctions/SJs from 4 patients at diagnosis and relapse	Source ddPCR data are available via the Source data Excel spreadsheet: Main figures
5f	BCP-ALL patient samples from VIVO biobank	N = 10 (Relapse)<br>N = 6 (Non-relapse)	LAM-ESC data available from European Genome-phenome Archive (EGA), under the dataset ID: EGAD50000000597.

			Processed data available from Supplementary Table 3.
Extended Data Fig. 1c *RAG1* expression upper	BCP-ALL patient samples from VIVO biobank	N = 44 (Relapse)<br>N = 77 (Non-relapse)	Source qPCR data are available via the Source data Excel spreadsheet: Extended Data
Extended Data Fig. 1c *RAG2* expression lower	BCP-ALL patient samples from VIVO biobank	N = 50 (Relapse)<br>N = 42 (Non-relapse)	Source qPCR data are available via the Source data Excel spreadsheet: Extended Data
Extended Data Fig. 1d left – *RAG1* expression	BCP-ALL patient samples from VIVO biobank	N = 44 (Relapse)<br>N = 77 (Non-relapse)	Source qPCR data are available via the Source data Excel spreadsheet: Extended Data
Extended Data Fig. 1d right – *RAG2* expression	BCP-ALL patient samples from VIVO biobank	N = 50 (Relapse)<br>N = 42 (Non-relapse)	Source qPCR data are available via the Source data Excel spreadsheet: Extended Data
Extended Data Fig. 2b	BCP-ALL patient samples from Charles University, Prague, Czech Republic	N = 6	
Extended Data Fig. 2c	BCP-ALL patient samples from HMDS	N = 3	
Extended Data Fig. 2d	BCP-ALL patient samples from HMDS. Samples and DNA were stored at 4°C	N = 3	Source ddPCR data are available via the Source data Excel spreadsheet: Extended Data
Extended Data Fig. 2e	*ETV6::RUNX1+* ALL patient WGS, described in Reference 19	N = 61	European Genome-phenome Archive (EGAD00001000116)
Extended Data Fig. 4a	BCP-ALL patient samples from VIVO biobank	N = 27	LAM-ESC data available from European Genome-phenome Archive (EGA), under the dataset ID: EGAD50000000597.<br>Processed data available from Supplementary Table 3.<br>Source ddPCR data are available via the Source data Excel spreadsheet: Extended Data
Extended Data Fig. 4b	BCP-ALL patient samples from VIVO biobank (Relapse and Non-relapse)<br>Healthy blood (University of Leeds)	N = 12 SJs from 7 patients (Relapse) and 10 patients (Non-relapse)<br>N = 2 (Healthy blood)	Source ddPCR data are available via the Source data Excel spreadsheet: Extended Data
Extended Data Fig. 4c	BCP-ALL patient samples from VIVO biobank	N = 26 for RAG1/SJ and	LAM-ESC data available from European Genome-phenome Archive (EGA), under the dataset ID: EGAD50000000597.

		N = 36 for RAG2/SJ comparisons	Processed data available from Supplementary Table 3.<br>Source qPCR data are available via the Source data Excel spreadsheet: Extended Data
Extended Data Fig. 4d	BCP-ALL patient samples from VIVO biobank	N = 6 patients (7 SJ/ recombination pairs)	Source ddPCR data are available via the Source data Excel spreadsheet: Extended Data
Extended Data Fig. 4e	BCP-ALL patient samples from VIVO biobank	N = 23 (SJ, Relapse); N = 26 (SJ, Non-relapse); N = 25 (REC, Relapse); N = 26 (REC, Non-relapse)	Source qPCR data are available via the Source data Excel spreadsheet: Extended Data
Extended Data Fig. 4e	BCP-ALL patient samples from VIVO biobank	N = 22 (SJ, Relapse); N = 23 (SJ, Non-relapse); N = 25 (REC, Relapse); N = 26 (REC, Non-relapse)	Source qPCR data are available via the Source data Excel spreadsheet: Extended Data
Extended Data Fig. 5a	BCP-ALL patient samples from VIVO biobank	N = 11 matched sets of SJs	LAM-ESC data available from European Genome-phenome Archive (EGA), under the dataset ID: EGAD50000000597.<br>Processed data available from Supplementary Table 3.
Extended Data Fig. 5b	BCP-ALL patient samples from VIVO biobank	N = 4 (patients with high SJ levels)<br>N = 3 (patients with low SJ levels)	RNA-seq data available from European Genome-phenome Archive (EGA) under the Accession Code EGAS00001006863.
Extended Data Fig. 6	BCP-ALL patient samples from VIVO biobank	N = 35 SJs that map to *IGK*<br>N = 20 SJs that map to *IGL*	LAM-ESC data available from European Genome-phenome Archive (EGA), under the dataset ID: EGAD50000000597.<br>Processed data available from Supplementary Table 3.
Extended Data Fig. 7a	BCP-ALL patient samples from VIVO biobank	N = 4 patients with high (right) or low (left) numbers of LAM-Recombination reads	LAM-Recombination data available from European Genome-phenome Archive (EGA), under the dataset ID: EGAD50000000597.<br>Processed data available from Supplementary Table 2.
Extended Data Fig. 7b	BCP-ALL patient samples from VIVO biobank	N = 3	Amplicon sequencing data are available from European Genome-phenome Archive (EGA), under the dataset ID: EGAD50000001518.
Extended Data Fig. 7c	BCP-ALL patient samples from VIVO biobank	N = 3 SJs from 3 patients	Source ddPCR data are available via the Source data Excel spreadsheet: Extended Data
Extended Data Fig. 7d	BCP-ALL patient samples from VIVO biobank	N = 77 recently generated SJs	LAM-recombination and LAM-ESC data, available from European Genome-phenome Archive (EGA), under the dataset ID: EGAD50000000597.<br>Processed data available from Supplementary Tables 2 and 3.

			Source ddPCR data (Fig. 3c) are available via the Source data Excel spreadsheet
Extended Data Fig. 7f	BCP-ALL patient samples from VIVO biobank	N = 3 patients	Source ddPCR data are available via the Source data Excel spreadsheet: Extended Data
Extended Data Fig. 8a	BCP-ALL patient samples from HMDS	N ≥ 3 technical repeats of 3 patient samples (patients M, N and P)	Source ddPCR data are available via the Source data Excel spreadsheet: Extended Data
Extended Data Fig. 8b	BCP-ALL patient samples from HMDS	N = 127, 101, and 108 images from 3 patients (M, N and P, respectively)	FISH data are available via Research Data Leeds: https://doi.org/10.5518/1693
Extended Data Fig. 8c	BCP-ALL patient samples from HMDS	N = 29, 24 and 26 DAPI-stained images and N = 127, 101 and 108 interphase FISH images from 3 patients (M, N and P, respectively)	FISH data are available via Research Data Leeds: https://doi.org/10.5518/1693
Extended Data Fig. 8d	BCP-ALL patient samples from HMDS	N = 2 images from 1 patient	FISH data are available via Research Data Leeds: https://doi.org/10.5518/1693
Extended Data Fig. 8e, f, g	BCP-ALL patient samples from VIVO biobank	N = 1585 total SJs from 71 patients	LAM-ESC data available from European Genome-phenome Archive (EGA), under the dataset ID: EGAD50000000597.<br>Processed data available from Supplementary Table 3.
Extended Data Fig. 9a	TARGET database; BCP-ALL patients	N = 150 patient samples at diagnosis	https://www.cancer.gov/ccg/research/genome-sequencing/target, dbGaP Sub-study ID: phs000464
Extended Data Fig. 9b,c	BCP-ALL patient samples from VIVO biobank	N = 12 (low SJ/ low *RAG1* expression)<br>N = 15 (low SJ/ high *RAG1* expression)<br>N = 14 (High SJ/ high *RAG1* expression	Source qPCR data are available via the Source data Excel spreadsheet: Extended Data. LAM-ESC data available from European Genome-phenome Archive (EGA), under the dataset ID: EGAD50000000597.<br>Processed data available from Supplementary Table 3.
Extended Data Fig. 9d	BCP-ALL patient samples from VIVO biobank	N = 12 (low SJ/ low *RAG1* expression); 5 WGS and 7 WES analysed<br>N = 15 (low SJ/ high *RAG1* expression); 5 WGS and 10 WES analysed<br>N = 14 (High SJ/ high *RAG1* expression; 8	WGS data available from European Genome-phenome Archive (EGA) under the Accession Code EGAS00001006863.<br>Whole exome sequencing data available from European Genome-phenome Archive (EGA), under the dataset ID: EGAD50000001519.

		WGS and 6 WES analysed	
Extended Data Fig. 9e	BCP-ALL patient samples from VIVO biobank		SVs examined in WGS data from samples at diagnosis - available from European Genome-phenome Archive (EGA) under the Accession Code EGAS00001006863. This was compared to SVs in WES data from samples at relapse, available from European Genome-phenome Archive (EGA), under the dataset ID: EGAD50000001519.<br>SVs were identified that are present at relapse at a single cRSS that were not present at diagnosis
Extended Data Fig. 10a	BCP-ALL patient samples from VIVO biobank	N = 27 matched LAM-Recombination reads at diagnosis and relapse from 9 patients	LAM-Recombination data available from European Genome-phenome Archive (EGA), under the dataset ID: EGAD50000000597.<br>Processed data available from Supplementary Table 2.
Extended Data Fig. 10b	BCP-ALL patient samples from VIVO biobank	N = 10 SJs (expansion)<br>N = 12 SJs (no expansion) from 8 patients	Source ddPCR data are available via the Source data Excel spreadsheet: Extended Data
Extended Data Fig. 10c	BCP-ALL patient samples from VIVO biobank	N = 5 DUX4-r patient samples	LAM-ESC data available from European Genome-phenome Archive (EGA), under the dataset ID: EGAD50000000597.<br>Processed data available from Supplementary Table 3.
Extended Data Fig. 10d	BCP-ALL patient samples from VIVO biobank	N = 4 SJs that appear to be circular<br>N = 4 SJs that appear to have integrated into the genome	Source ddPCR data are available via the Source data Excel spreadsheet: Extended Data
Extended Data Fig. 10e	BCP-ALL patient samples from VIVO biobank	high hyperdiploidy patients N = 15<br>*TCF3::PBX1* patients N = 5<br>*PAX5* patients N = 8	LAM-ESC data available from European Genome-phenome Archive (EGA), under the dataset ID: EGAD50000000597.<br>Processed data available from Supplementary Table 3.
